# Environmental Exposure of the Mouse Germ Line: DNA Adducts in Spermatozoa and Formation of *De Novo* Mutations during Spermatogenesis

**DOI:** 10.1371/journal.pone.0011349

**Published:** 2010-06-28

**Authors:** Ann-Karin Olsen, Åshild Andreassen, Rajinder Singh, Richard Wiger, Nur Duale, Peter B. Farmer, Gunnar Brunborg

**Affiliations:** 1 Division of Environmental Medicine, Norwegian Institute of Public Health, Oslo, Norway; 2 Department of Cancer Studies and Molecular Medicine, University of Leicester, Leicester, United Kingdom; UMDNJ, United States of America

## Abstract

**Background:**

Spermatozoal DNA damage is associated with poor sperm quality, disturbed embryonic development and early embryonic loss, and some genetic diseases originate from paternal *de novo* mutations. We previously reported poor repair of bulky DNA-lesions in rodent testicular cells.

**Methodology/Principal Findings:**

We studied the fate of DNA lesions in the male germ line. B[a]PDE-N^2^-dG adducts were determined by liquid chromatography-tandem mass spectrometry, and *de novo* mutations were measured in the *cII*-transgene, in Big Blue®mice exposed to benzo[a]pyrene (B[a]P; 3×50 mg/kg bw, i.p.). Spermatozoa were harvested at various time-points following exposure, to study the consequences of exposure during the different stages of spermatogenesis. B[a]PDE-N^2^-dG adducts induced by exposure of spermatocytes or later stages of spermatogenesis persisted at high levels in the resulting spermatozoa. Spermatozoa originating from exposed spermatogonia did not contain DNA adducts; however *de novo* mutations had been induced (p = 0.029), specifically GC-TA transversions, characteristic of B[a]P mutagenesis. Moreover, a specific spectrum of spontaneous mutations was consistently observed in spermatozoa.

**Conclusions/Significance:**

A temporal pattern of genotoxic consequences following exposure was identified, with an initial increase in DNA adduct levels in spermatozoa, believed to influence fertility, followed by induction of germ line *de novo* mutations with possible consequences for the offspring.

## Introduction

Germ cells are unique, since they transmit their genetic information to the next generation. Progenitor male germ cells carrying DNA damage have the potential to produce spermatozoa that, upon fertilization, may give rise to offspring with compromised health. With respect to fertility as many as one in twenty sexually mature men is considered infertile [1], and there is strong evidence that sperm counts in men living in industrialized countries are low and declining [2]. Spermatozoa from infertile men exhibit higher levels of DNA damage compared to fertile men, and sperm DNA damage is strongly associated with low sperm quality [3]–[8] but also reduced fertility [9], perturbed foetal development and early embryonic loss [10]. Paradoxically, spermatozoa with extensive DNA damage retain their capacity to fertilize oocytes [10]. This study is part of an integrated effort to understand the implications for fertility and the health of the offspring, of exposure of the male to an ubiquitous environmental agent, benzo[a]pyrene (B[a]P).

During the strictly timed and regulated process of spermatogenesis, stem cell spermatogonia develop into mature spermatozoa, involving mitotic, meiotic, and post-meiotic phases. The spermatogonial stem cells pass through several mitotic divisions after which they give rise to primary spermatocytes which enter meiosis; alternatively, the stem cells continue to divide as spermatogonia thereby maintaining the stem cell pool. The spermatocytes undergo synapsis, meiotic recombination and two rounds of cellular division to generate haploid spermatids which - after cellular differentiation including extensive chromatin condensation–ultimately become spermatozoa. It is to be expected that the susceptibility to DNA damage induction and mutation fixation would vary considerably during these processes. Many genetic disorders in the offspring are due to mutations pre-existing in the germ-line; however, as many as ∼20% arise as *de novo* mutations [11], [12]. For several autosomal dominant disorders such as achondroplasia, the Apert and Waardenburg syndrome [12], [13], the mutations are almost exclusively of paternal origin. Nucleotide substitutions and small insertions/deletions representing two of the most common types of mutations causing known genetic disease [14] are most frequent among paternal mutations; such genetic changes are associated with replication errors during the higher number of cell divisions taking place in the male versus the female germ line [15].

DNA damage may be removed via various DNA repair mechanisms, including nucleotide- (NER) and base-excision repair (BER). Both NER and BER are characterized by a “cut-synthesize-paste mode of action. Generally they remove different classes of DNA damage, but for some DNA lesions redundancies exist, i.e. these lesions may be removed via several DNA repair mechanisms. We previously reported that testicular cells rather unexpectedly exhibit limited or no NER of some bulky DNA adducts [16], [17]. One may therefore speculate that germ cells could be specifically susceptible to genotoxic chemicals which cause some types of bulky DNA adducts. Possible outcomes of exposure to such chemicals are; i) The affected male germ cell may be eliminated, ii) the cell may survive but the resulting spermatozoon carries DNA adducts; iii) *de novo* mutations are formed.

Poor NER in male germ cells warranted studies of the fate of DNA lesions induced in male germ cells, and whether residing DNA lesions could induce *de novo* mutations. We used B[a]P as a model compound since it represents a chemical that humans are ubiquitously exposed to, via e.g. cigarette smoke, diesel exhaust and charbroiled foods. As a reproductive genotoxicant, B[a]P induces dominant lethal mutations in post-meiotic mouse germ cells (early and mid spermatozoa) but not heritable translocations [18]. B[a]P generates adducts in DNA mainly via the benzo[a]pyrene diol epoxide (B[a]PDE) metabolite that reacts with the N^2^-exocyclic amino group of deoxyguanosine to form 10-(deoxyguanosin-N^2^-yl)-7,8,9-trihydroxy-7,8,9,10-tetrahydrobenzo[a]pyrene (B[a]PDE-N^2^-dG), a bulky adduct mainly repaired via NER.

We here report on the genotoxic susceptibility of different stages of spermatogenesis in wild-type mice treated with B[a]P. Levels of B[a]PDE-N^2^-dG adducts were measured and *de novo* mutations were measured and characterised, in epididymal spermatozoa originating from male germ cells that were in specific stages of spermatogenesis at the time of exposure.

## Materials and Methods

### Animals

Big Blue® homozygous mice (C57BL/6) from Stratagene (Texas, US), bred in-house, were housed in plastic cages with 12 h light/dark cycle, controlled humidity (55±5%), temperature (20–24°C), and water *ad libitum*. Mice were given standard chow; breeding diet, SDS RM3 (E), during gestation and until weaning, and thereafter a standard maintenance diet, SDS RM1 (E), (Special Diets Services, Witham, UK). The experiments were approved by the National Experimental Animal Board of Norway (DVM PhD Dag M. Eide; project 269). Male mice (8–10 weeks) were exposed intraperitoneally (i.p.) to 50 mg B[a]P (≥96%, B1760, Sigma-Aldrich, dissolved in corn oil) per kg bodyweight on days 0–2, i.e. a total of 150 mg per kg bodyweight. The first day of exposure was defined as day 0. Untreated mice (Day 0) and mice exposed to corn oil were used as controls. Mice were asphyxiated and sacrificed using CO_2_ at day 0 (untreated) and at day 4, 16, 30, 44 or 119 after the first exposure. The experiments were designed to attain specific information about male germ cells exposed at different stages of spermatogenesis, assuming that exposure to B[a]P did not affect progression of spermatogenesis. Epididymal spermatozoa were collected and liver was used as somatic tissue for comparison. Testes and liver were weighed and quickly frozen on dry ice.

### Preparation and characterization of epididymal spermatozoa

The testes were weighed, and testicular cell suspensions were prepared from one decapsulated testicle [19]. The epididymis was dissected into caput, cauda and vas deferens, and weighed. The relative organ weights were calculated by dividing the organ weights by the body weight of each animal. Cauda spermatozoa were recovered following 2–4 cuts into fresh tissue, or from vas deferens, by squeezing the cells into medium. Tissue fragments were allowed to settle and the supernatant was filtered (165 µm nylon filter). Caput spermatozoa were prepared similarly from frozen tissue. One aliquot of each sample was fixed in 0.2% paraformaldehyde in PBS to determine its purity by flow cytometric analyses. The other cells were pelleted, flash frozen in dry ice and stored at −80°C. Cell distributions were determined by flow cytometry as described earlier [20], [21]. The percentages of cells in the 1C, 2C, S-phase and 4C populations were estimated from DNA cytograms using the Multicycle Program (Phoenix Flow System, San Diego, CA, USA). The progression of spermatogenesis is considered normal if there are >70% 1C cells and if the cell suspension consists of cells of different ploidy in the relationship 1C>4C>2C [22].

### Determination of B[a]PDE-N^2^-dG adducts by liquid chromatography–tandem mass spectrometry

Genomic DNA was isolated from caput spermatozoa or liver using the Blood & Cell Culture DNA Midi Kit (Qiagen AB, Sweden) as previously described [23]. For caput spermatozoa a loose fitting pestle was used for homogenization and 2 mM CaCl_2_ and 20 µl/ml β-mercaptoethanol were added during digestion. DNA concentrations and purities were determined using a NanoDrop Spectrophotometer (Thermo Scientific, Waltham, MA). The levels of B[a]PDE-N^2^-dG adducts were determined in DNA samples (4–8 individual mice per group) from cauda spermatozoa (22–50 µg) or liver (27–50 µg) using positive electro spray ionization liquid chromatography–tandem mass spectrometry (LC–MS/MS) selected reaction monitoring (SRM) with the incorporation of a [^15^N_5_]-stable isotope labelled B[a]PDE-N^2^-dG internal standard as previously described [23].

### Big Blue® Mouse *cII*-Mutation Assay

Genomic DNA from cauda spermatozoa was isolated as described in the Big Blue DNA Isolation kit (Stratagene, La Jolla, CA, USA) with adaptations; β-mercaptoethanol (40 µl/ml) and CaCl_2_ (4 mM) was added during proteinase K digestion (up to 3 h). DNA was extracted by phenol-chloroform and precipitated/concentrated using 100% ethanol. When the DNA was too viscous to precipitate, it was collected using a genomic tip. The DNA was concentrated by filter column centrifugation (Ultrafree-MC, cut off at 30 KDa, Millipore) followed by dialysis on filters (HAWPO 4700, Millipore) floating on dialysis buffer for 16 h. Liver genomic DNA was extracted using the RecoverEase DNA Isolation Kit (Stratagene, La Jolla, CA, USA) as recommended, except that dialysis was carried out as described above.

Mutation frequencies (Mf) were measured using the lambda Select-*cII* Mutation Detection System of Big Blue® mice as recommended, with modifications. Data from packaging reactions with <200,000 plaque-forming units (pfu) were not included. The λ transgenic shuttle vector recovery was performed as described by Stratagene's Transpack Packaging Extracts producing packaged DNA samples as λ-phages, with adaptations for cauda spermatozoa; 8-15 µl DNA was packaged, and when necessary incubation was prolonged (with up to 2 h) and/or additional packaging extract was added. The λ-phages were adsorbed to G1250 *E. coli*, mixed with agar and poured onto Petri dishes that were incubated at 37°C for 24 h (titer; total number of pfu) or at 24°C for 48 h (mutants). Putative mutants were confirmed by replating. The *cII* Mf is calculated as the number of mutant *cII* pfu (24°C) divided by the total pfu screened (37°C).

### Sequencing of mutants

Mutant DNA was amplified by PCR and sequenced in both directions, as recommended by Stratagene for the λ Select-*cII*™ mutation detection system for Big Blue® rodents (Stratagene, CA, USA). The PCR conditions for amplification were 95°C for 10 min, followed by 35 cycles (94°C for 15 sec, 56°C for 15 sec and 72°C for 20 sec) and a final extension of 72°C for 5 min. The PCR products were sequenced as previously described [24] using the recommended primers at the following conditions: 95°C for 10 min, followed by 30 cycles (95°C for 30 s, 60°C for 1 min and 72°C for 2 min) and a final extension of 72°C for 10 min. Alternatively, mutants were sequenced by Cogenics (UK). The DNA sequences were assembled and compared to consensus sequence with the CEQ 8000™ Genetic Analysis System version 8.0.52 (Beckman Coulter™) or SeqScape Sequence Software version 2.5.0 (Applied Biosystems, CA, USA). Identical mutations in each animal were considered clonal, and excluded; the prevalent frameshift mutation due to insertion of one guanine (InsG) in the G-run in codons 60–62 was thus counted as one single mutation per mouse. Intragenic suppressor mutations were considered separate events, and calculated as such.

### Statistics

Differences in bodyweight, relative weights of organs, B[a]PDE-N2-dG adducts and mutation frequencies in both testis and liver were analysed by Levene's test for homogeneity of variances, one-way analysis of variance (ANOVA) followed by a post-hoc least significant difference (LSD) test to allow multiple comparisons when appropriate; alternatively a non-parametric, Mann-Whitney U test was used. The method for each comparison is stated in the Results and in Figures and Tables. Comparisons were made between B[a]P-exposed samples at the different time points and untreated control and between B[a]P-exposed and time-matched corn oil-treated mice sacrificed at 119 days after exposure. The analyses were performed using the SPSS software (SPSS, Inc., Chicago, IL) and p<0.05 was considered significant. Differences in frequencies of different classes (transversions, insertions, insertions and deletions) or types (GC-TA, InsG, CG-TA) of mutations were tested using the hypergeometric test. Differences in mutation spectra were tested using the method of Adams and Skopek based on 20,000 iterations [25].

## Results

The study was designed to obtain specific information about the susceptibilities of male germ cells at different stages of spermatogenesis to the genotoxic effects of B[a]P (3×50 mg/kg bw, i.p.) [26]. This was achieved by exposing mice and then leaving them for various periods of time (0–119 days) before sacrifice. The caput and cauda epididymal spermatozoa analysed at the various time points after exposure thus emanate from germ cells exposed at different stages of spermatogenesis; spermatogonial stem cells at 119 days, differentiating spermatogonia at 44 days, spermatocytes at 30 days, spermatids at 16 days and epididymal spermatozoa at 4 days after exposure, along with spermatozoa from untreated mice (day 0). DNA adducts and mutations were measured in caput and cauda spermatozoa, respectively. These measurements were preferably done in the same animal (consistently for the 119-day samples), allowing direct comparison of outcomes. We considered the stem cell spermatogonia as the most important cell type to be studied with respect to mutation analysis since such mutations will be present in every spermatozoon generated from these progenitor cells, whereas mutations arising in the later stages of spermatogenesis are temporary and will affect only very few spermatozoa.

Minor changes in body and testicular weight developments were noted but did not indicate any significant systemic or reproductive organ toxicity from the B[a]P treatments (Supplementary Figure S1), and the progression of spermatogenesis was not significantly disturbed by exposure to B(a)P (data not shown), as measured by evaluating distributions of testicular cell samples by flow cytometry [22].

### B[a]PDE-N^2^-dG adduct formation

**Figure 1:**
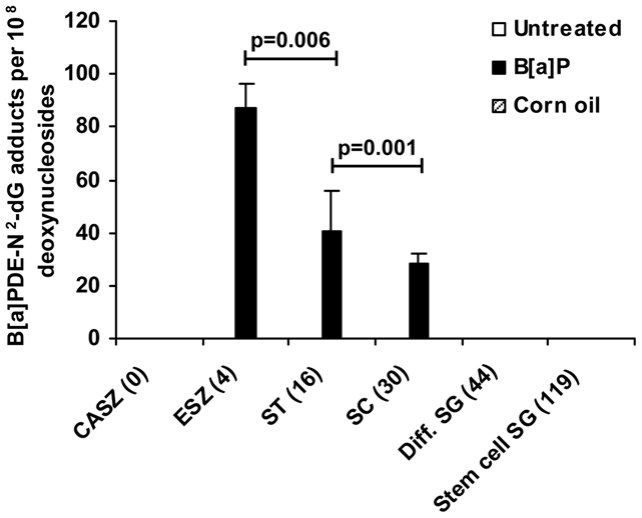
B[a]PDE-N^2^-dG adducts in caput spermatozoa from untreated and exposed male germ cells at different stages of spermatogenesis. The male germ cell stage is indicated along with day of sacrifice. Untreated cauda spermatozoa = CASZ (number of animals n = 4); epididymal spermatozoa = ESZ (n = 4); spermatids = ST (n = 4); spermatocytes = SC (n = 6); differentiating spermatogonia = Diff. SG (n = 4); stem cell spermatogonia = Stem cell SG (n = 5). B[a]PDE-N^2^-dG adduct levels are given as means±SE. For untreated CASZ and exposed Diff. SG and Stem cell SG the values were below detection. Statistically significant differences and p-values are indicated in the figure (ANOVA and post hoc LSD).

B[a]PDE-N^2^-dG was determined in DNA isolated from caput spermatozoa originating from male germ cells exposed at different stages of spermatogenesis. The mean fraction of spermatozoa in these samples was 60.6±7.4%. B[a]PDE-N^2^-dG adducts introduced into spermatocytes or subsequent stages of spermatogenesis persisted throughout differentiation into caput spermatozoa, i.e. for at least 30 days post-exposure (Figure 1 and Supplementary Table S1). The exposure of the different cell stages resulted in varying levels of DNA adducts in the resulting caput spermatozoa. Measured per 10^8^ deoxynucleosides, the level of B[a]PDE-N^2^-dG adducts was highest in caput spermatozoa originating from exposed epididymal spermatozoa (4 days; 87.4±8.9; n = 4), whereas it was significantly lower in spermatozoa originating from exposed spermatids (16 days; 40.7±15.2; n = 4; p = 0.006, ANOVA and post-hoc LSD) or from exposed spermatocytes (30 days; 28.5±3.8; n = 6; p = 0.001, ANOVA and post-hoc LSD). As opposed to meiotic and post-meiotic male germ cells, no detectable B[a]PDE-N^2^-dG adducts were observed in caput spermatozoa originating from exposed differentiating (n = 4) or stem cell spermatogonia (n = 5), or corn oil exposed spermatogonia.

**Figure 2:**
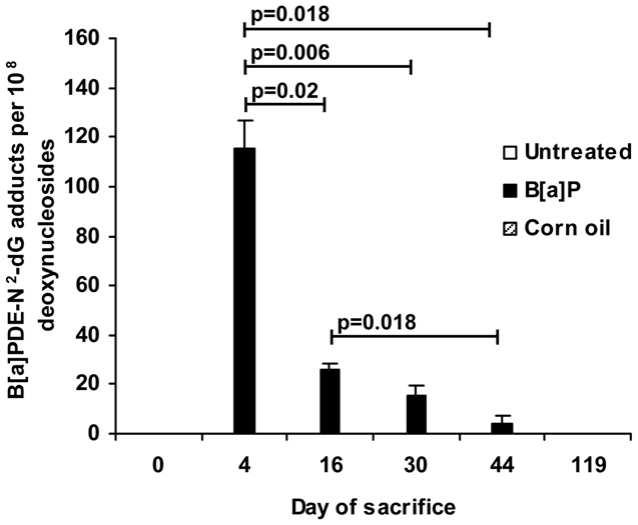
B[a]PDE-N^2^-dG adducts in liver from untreated and exposed mice. The day of sacrifice is indicated for untreated and exposed mice. B[a]PDE-N^2^-dG adduct levels from 4–8 mice per group are given as means±SE. Reduction in B[a]PDE-N^2^-dG adduct levels were tested statistically (Mann-Whitney) between groups and are indicated with p-values in the figure.

In the liver (Figure 2, Supplementary Table S2), the level of B[a]PDE-N^2^-dG adducts peaked at day 4 after exposure (115.5±11.2, n = 4), and showed a significant decline (Mann-Whitney) with time at 16 days (26.0±2.5, p = 0.021, n = 4), 30 days (15.4±4.1; p = 0.006, n = 4) and 44 days (3.7±3.7; p = 0.018, n = 8) to non-detectable levels at 119 days after exposure (n = 5 for both).

### *De novo* mutations

**Figure 3:**
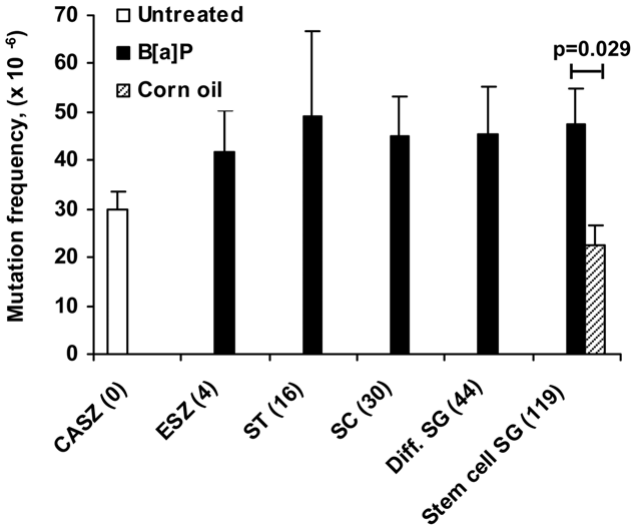
Mutation frequencies in cauda spermatozoa from B[a]P-exposed male germ cells at different stages of spermatogenesis. The male germ cell stage is indicated along with day of sacrifice. Cauda spermatozoa = CASZ (untreated, n = 3); epididymal spermatozoa = ESZ (n = 4); spermatids = ST (n = 6); spermatocytes = SC (n = 5); differentiating spermatogonia = Diff. SG (n = 6); stem cell spermatogonia = Stem cell SG (n = 6). Mf values are given as means (per 10^6^ pfu) ±SE. Differences in mean Mf were tested statistically (Mann-Whitney); The B[a]P-exposed were compared with untreated control, and B[a]P-exposed at 119 days were also compared with its time-matched corn oil treated control. Statistically significant differences are indicated (p = 0.029).

**Figure 4:**
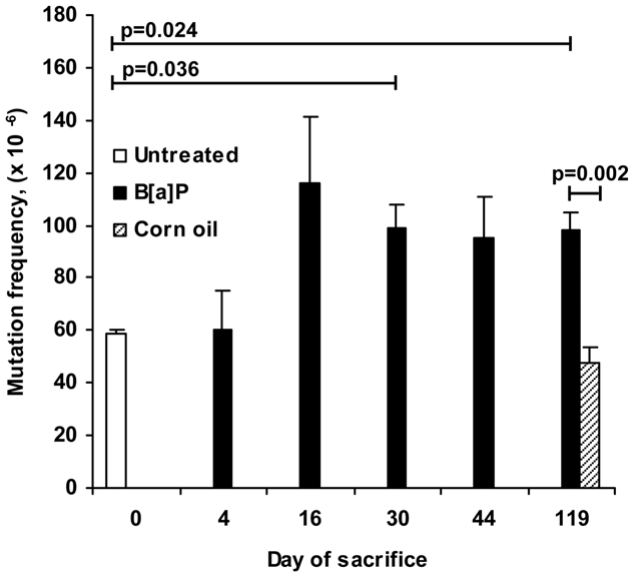
Mutation frequencies in the liver of Big Blue® mice exposed to B[a]P. The Mf values are given as means (per 10^6^ pfu) ±SE, and the days of sacrifice are indicated on the x-axis. Mice sacrificed at day 0 are untreated controls. Treatments groups and number of mice (n) are given: 0 days untreated (n = 3); 4 days B[a]P (n = 4); 16 days B[a]P (n = 6); 30 days B[a]P (n = 5); 44 days B[a]P (n = 6); 120 days B[a]P (n = 6) and 120 days corn oil (n = 6). Statistically significant differences (Mann-Whitney) between the Mf of the groups and p-values are indicated.

Induction of *de novo* mutations was measured in genomic DNA from cauda spermatozoa, containing 96–99% spermatozoa, or from liver. For cauda spermatozoa a total of 15 million plaque forming units (pfu) were screened and 440 mutants were isolated. From livers, 33 million pfu were screened and 2146 mutants were isolated. In untreated mice the mean mutation frequency (Mf) in cauda spermatozoa (29.7±3.7×10^−6^, n = 6) was significantly lower than that of the liver (58.5±1.9×10^−6^, n = 3; p = 0.024 (Mann-Whitney), Figures 3 and 4, and Tables 1 and 2). B[a]P-exposure resulted in approximately two-fold higher mean Mf in cauda spermatozoa from all stages of spermatogenesis tested compared to untreated controls (Figure 3 and Table 1), but these differences were not significant when compared to untreated control. The mean Mf was however significantly increased (p = 0.029, Mann-Whitney) for cauda spermatozoa derived from B[a]P-exposed stem cell spermatogonia (47.7±7.1×10^−6^, n = 4), compared to corn oil treated age-matched samples of stem cell spermatogonia (22.5±4.0×10^−6^, n = 4; Figure 3, Table 1). The Mf of the liver increased approximately two-fold at 16 days after exposure and was maintained at this level, relative to corn oil treated mice (47.9±6.0×10^−6^, n = 6; p = 0.015 (ANOVA and post-hoc LSD) Figure 4, Table 2), up to 119 days after B[a]P-exposure.

**Table 1:** Mutation frequencies in caput spermatozoa.

Caput spermatozoa								
Exposed spermatogenic cell type	Day of sacrifice	Treatment	n	Pfu* screened	Mutants isolated	Mf×10^−6^	Mean of Mf×10^−6^	SE
Cauda spermatozoa	0	None	6	300 200	4	16.8	29.7	3.7
				748 800	13	22.2		
				497 683	14	36.2		
				425 646	9	27.1		
				651 206	20	39.8		
				501 749	14	36.1		
Epididymal spermatozoa	4	B[a]P	3	598 276	13	28.0	41.7	8.7
				717 237	22	39.4		
				672 557	30	57.7		
Spermatids	16	B[a]P	2	560 280	29	66.7	49.0	17.8
				331 911	8	31.2		
Spermatocytes	30	B[a]P	5	656 236	9	17.7	45.0	8.2
				644 000	22	44.0		
				318 636	10	39.9		
				382 862	18	60.8		
				227 136	11	62.7		
Differentiating spermatogonia	44	B[a]P	3	517 132	2	54.8	45.5	9.8
				546 322	11	26.0		
				255 242	11	55.8		
Stem cell spermatogonia	119	B[a]P	4	637 560	30	60.6	47.7**	7.1
				806 369	20	31.9		
				578 634	18	39.6		
				815 304	37	58.5		
Stem cell spermatogonia	119	Corn oil	4	702 733	13	23.8	22.5	4.0
				627 256	13	26.7		
				718 704	16	28.7		
				348 726	3	10.9		

*Pfu = plaque forming unit.

**Statistically different compared to corn oil treated time-matched mice (p = 0.029, Mann-Whitney).

**Table 2:** Mutation frequencies in the liver.

Liver							
Day of sacrifice	Treatment	n	Pfu* screened	Mutants isolated	Mf×10^−6^	Mean of Mf×10^−6^	SE
0	None	3	1 232 505	57	59.1	58.5	1.9
			391 495	19	61.5		
			1 192 515	51	54.9		
4	B[a]P	4	1 241 600	44	45.4	60.1	15.3
			1 054 080	30	36.4		
			1 094 400	46	53.8		
			386 740	32	104.9		
16	B[a]P	6	1 051 200	145	176.6	109.9	25.6
			1 098 880	44	51.3		
			1 148 160	107	119.3		
			672 960	45	85.6		
			1 222 080	103	107.9		
			871 680	81	118.9		
30	B[a]P	5	1 779 380	134	96.3	98.7**	9.1
			1 212 800	79	83.4		
			1 237 120	100	103.5		
			603 560	38	79.6		
			223 485	23	130.5		
44	B[a]P	6	504 000	65	165.1	95.0	15.5
			884 160	67	97.0		
			543 360	23	54.2		
			1 081 920	61	72.2		
			529 920	35	83.5		
			452 160	35	97.8		
119	B[a]P	6	1 422 400	135	121.5	98.1***, †	7.1
			982 080	82	105.5		
			785 280	46	75.0		
			1 537 600	100	83.2		
			802 010	69	109.1		
			1 122 055	83	94.4		
119	Corn oil	6	1 563 200	44	36.0	47.9	6.0
			619 200	23	47.5		
			651 435	32	62.3		
			366 135	14	48.5		
			984 000	21	27.3		
			637 170	33	65.7		

*Pfu = plaque forming unit.

** = Statistically different from untreated control, p = 0.036 (Mann-Whitney).

*** = Statistically different from untreated control, p = 0.024 (Mann-Whitney).

† = Statistically different from corn oil exposed time-matched control, p = 0.002 (Mann-Whitney).

### Mutation spectra

The mutants from untreated cauda spermatozoa, cauda spermatozoa originating from stem cell spermatogonia (119 days, both B[a]P- and corn oil-exposed), and the corresponding liver mutants, were characterized by DNA sequencing to establish mutation patterns. The sequences of a total of 181 mutants from cauda spermatozoa are presented (Table 3), of which 55 were from untreated mice, 65 from B[a]P-exposed mice, and 61 from corn oil treated mice. A described in Materials and Methods, specific mutations at the same base position occurring more than once per animal was considered as one single mutational event. Following exclusion of such possibly clonal mutations in cauda spermatozoa there were 35 (untreated), 45 (B[a]P), and 52 (corn oil) mutations.

**Table 3:** Mutants characterised from cauda spermatozoa and liver.

		Total # of mutants sequenced	Frequency of InsG prior to clonal exclusion (%)	# mutants after clonal exclusion*	Frequency of InsG after clonal exclusion (%)	# of insertion of G excluded/total # of excluded mutants (%)**
Cauda spermatozoa	Untreated	55	43.6	35	14.3	19/20 (95)
	B[a]P	65	46.2	45	11.1	14/20 (70)
	Corn oil	61	23.0	52	9.6	9/9 (100)
	*Total*	*181*		*132*		
Liver	B[a]P	52	3.8	46	2.2	1/6 (16.7)
	Corn oil	58	3.4	46	4.3	0/11 (0)
	*Total*	*110*		*92*		

*Identical mutations at an identical position or within the G-run in codons 60–62 in the *cII*-gene occurring more than once in one animal are considered clonal and are excluded.

**The number of single insertions of G (and %) is indicated relative to the total number of clonally excluded mutants.

The large majority of the excluded mutations in spermatozoa in all three groups were a single insertion of a guanine (InsG) in a G-run of six guanines in codons 60–62 of *cII*. Within this G-run, the base position of the mutation cannot be defined, and all the InsGs are thus regarded as identical mutations; they will always be counted as one single mutation if occurring in the same animal. As many as 19 of 20 of the excluded mutations in cauda spermatozoa of untreated mice were InsGs (Table 3). For B[a]P-exposed and corn oil treated mice, 14 of 20 and 9 of 9 were InsGs, respectively. In the liver, markedly fewer putative clonal mutations (of all types) were excluded (15.5%, compared to 27.1% in cauda spermatozoa); these rarely involved InsG (1 of a total of 17; ∼6%).

Significantly different mutation patterns (with respect to frequencies/sites and locations) were observed in the spermatozoa originating from B[a]P-exposed stem cell spermatogonia, compared to corn oil treated age-matched control mice (p = 0.03, Adams and Skopek test, [25]). Such differences were not found when comparing B[a]P-exposed and untreated mice. In the liver the mutation patterns did not differ significantly between B(a)P- and corn oil treated mice.

**Figure 5:**
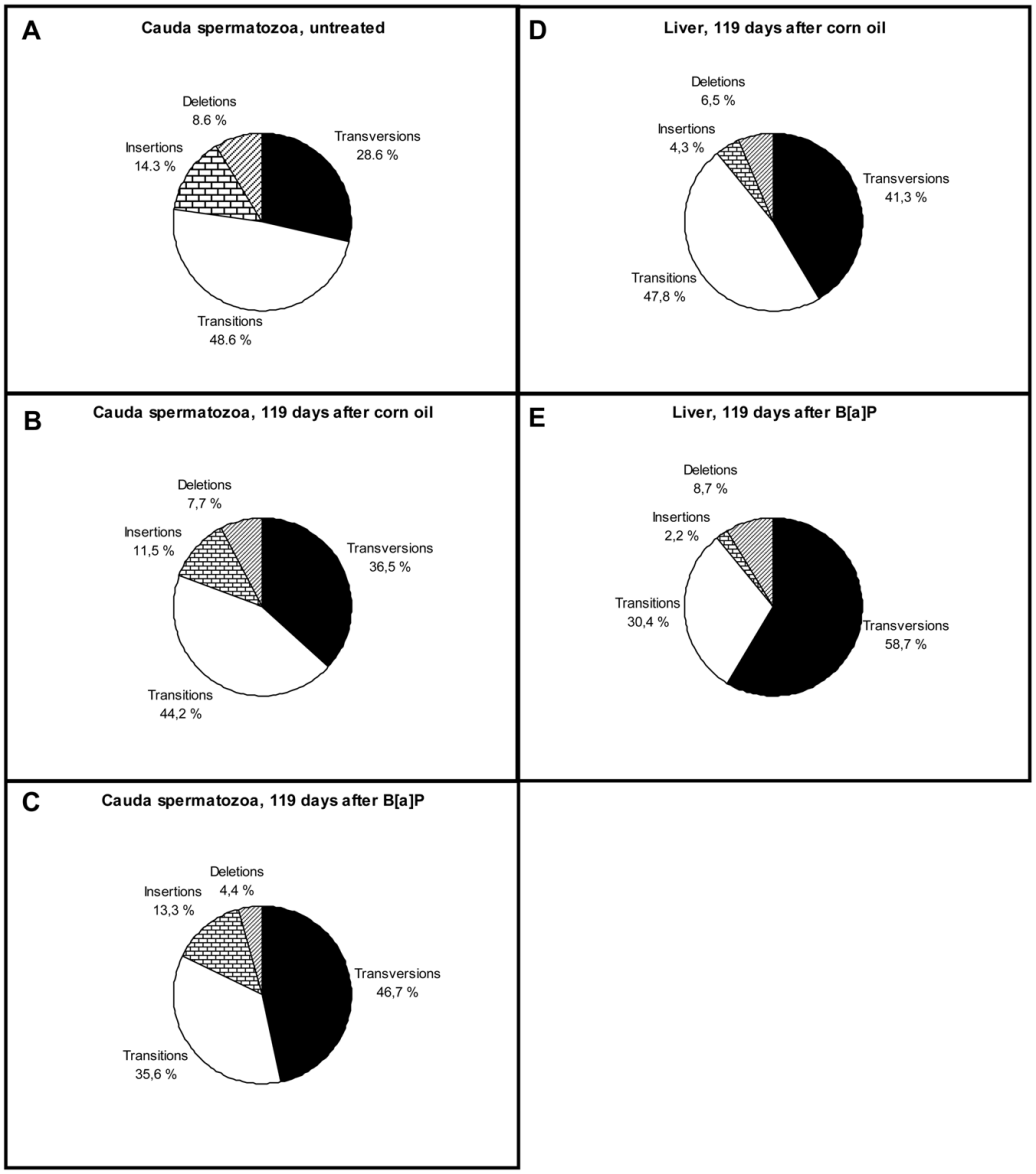
Mutation spectra in cauda spermatozoa and liver. The figure shows the distributions of mutation types from cauda spermatozoa of untreated mice (A), mice treated with corn oil (B), B[a]P (C) 119 days before sacrifice, or from livers of mice treated with corn oil (D) or B[a]P (E) 119 days before sacrifice. The distributions are given as percentages (%) of the total number of mutations.

The frequency of transversions in spermatozoa seemed to be increased by B[a]P; this increase was statistically significant compared to untreated mice (p = 0.048, hypergeometric test, Figure 5A and C) but not when compared to corn oil treated mice (p = 0.099; Figure 5B and C). More specifically, the frequency of GC-TA transversions in cauda spermatozoa (Table 4) increased from 17.3% (corn oil) to 33.3% (B[a]P, p = 0.037, hypergeometric test) whereas the increase from 22.8% in untreated mice was not statistically significant (p = 0.119). In the liver there were increased levels of GC-TA transversions (p = 0.001, hypergeometric test, 43.4% vs 13.3% for B[a]P and corn oil, respectively; Table 4).

**Table 4:** Distributions of mutation types in cauda spermatozoa and liver.

Class of mutants	Mutation	Cauda spermatozoa			Liver	
		Untreated # (%)	Corn oil (119 days after exposure) # (%)	B[a]P (119 days after exposure) # (%)	Corn oil (119 days after exposure) # (%)	B[a]P (119 days after exposure) # (%)
Transitions	**CG-TA**	11 (31.4)	20 (38.4)	11 (24.4)	20 (43.4)	14 (30.4)
	*CG-TA in CpG* *	*7 (63.6)*	*11 (55.0)*	*5 (45.5)*	*13 (65.0)*	*11 (84.6)*
	**AT-GC**	6 (17.1)	3 (5.7)	5 (11.1)	2 (4.3)	
Transversions	**GC-TA**	8 (22.8)	9 (17.3)	15 (33.3)	6 (13.1)	20 (43.4)
	**GC-CG**		5 (9.6)	2 (4.4)	6 (13.0)	4 (8.7)
	**AT-TA**	1 (2.9)	2 (3.8)	2 (4.4)	4 (8.6)	1 (2.2)
	**AT-CG**	1 (2.9)	3 (5.8)	2 (4.4)	3 (6.5)	2 (4.3)
Insertions	**G**	5 (14.3)	5 (9.6)	5 (11.1)	2 (4.3)	1 (2.2)
	**A**		1 (1.9)	1 (2.2)		
Deletions	**G**	3 (8.6)	3 (5.8)		1 (2.2)	2 (4.3)
	**C**			2 (4.4)	1 (2.2)	1 (2.2)
	**T**				1 (2.2)	
	**A**					1 (2.2)
	**CT** **		1 (1.9)			
Total		35 (100)	52 (100)	45 (100)	46 (100)	46 (100)

*The number of CG-TA transitions (and %) occurring in CpG dinucleotides.

**A double deletion of CT at codon 58 was considered one single mutational event.

The InsGs were particularly frequent in spermatozoa, irrespective of exposure. Prior to exclusion of possible clonal mutations, frequencies of InsGs in cauda spermatozoa were 6–14 times higher than in the liver (Table 3; in cauda spermatozoa within the range 23.0–46.2% and in liver 3.4–3.8%). For all treatment groups, few mice carried InsG mutations in the liver (3 out of 7), whereas almost all (13 of 16) had such mutations in spermatozoa. A consequence of the high prevalence of InsGs in spermatozoa, combined with the strict criteria for clonal exclusion, is that the frequency of InsGs in spermatozoa-after clonal exclusion -did not appear significantly different from the liver (hypergeometric test).

Intragenic suppressor mutations were observed after B[a]P-exposure only; there were three among the cauda spermatozoa mutants (AT-GC plus deletion of C, CG-AT plus GC-TA, and AT-GC plus CG-AT, in adjacent base positions) and two among the liver mutants (one double GC-TA and one GC-CG plus GC-TA). One deletion of two adjacent bases, CT, was observed among the spermatozoa mutants from corn oil treated mice.

Without B[a]P, the overall distribution of the types of mutations was similar (transitions>transversions>insertions>deletions; Figure 5A, B and D), in both untreated (spermatozoa) and corn oil treated (spermatozoa and liver) mice. The most prevalent base substitution, CG-TA transition, was less frequent in spermatozoa (31.4%, untreated; 38.4%, corn oil) than in liver (43.4%, corn oil) of which the majority occurred in CpG dinucleotides (Table 4).

## Discussion

This study presents a detailed analysis of the persistence of defined DNA adducts in spermatozoa following exposure to B[a]P at different stages of spermatogenesis, including the stem cell stages, along with stage specific analyses of *de novo* germ line mutations and characterization of mutants. In mice exposed to B[a]P, high numbers of B[a]PDE-N^2^-dG adducts persisted to the stage of spermatozoa when introduced into spermatocytes or later stages of spermatogenesis (Figure 1, Supplementary Table S1). The fertilizing spermatozoa thus contain significant levels of DNA adducts. DNA adducts induced in differentiating or stem cell spermatogonia, however, did not persist to spermatozoa. On the other hand, *de novo* mutations were significantly induced in stem cell spermatogonia (Figure 3, Table 1). Thus, exposure to B[a]P gives distinct and different consequences depending on the exposed spermatogenic cell stage. Important implications of our findings are that the mutation rate observed in spermatozoa reflects the exposure load acquired during the entire life span of an individual, whereas the DNA damage levels in spermatozoa are temporary and reflect recent exposures.

### DNA damage

The results clearly indicate that cells in different stages of spermatogenesis exhibit specific response patterns with respect to both induction and removal of DNA adducts from B[a]P (Figure 1, Supplementary Table S1). Exposure of stem cell or differentiating spermatogonia does not lead to B[a]PDE-N^2^-dG adducts persisting to the stage of spermatozoa.

With respect to meiotic and post-meiotic cells, B[a]PDE-N^2^-dG adducts were induced in spermatocytes, spermatids and epididymal spermatozoa since these adducts persisted to caput spermatozoa 30, 16 and 4 days after exposure, respectively. Since the initial adduct level in each stage could not be measured using our experimental design, differences in adduct levels between stages cannot be interpreted as reflecting DNA repair *per se*. Adducts are indeed induced in spermatogonia, as was also recently shown for spermatozoa originating from B(a)P-exposed spermatogonia in DNA repair deficient (XPC^−/−^) mice [27], implying that the adducts are removed when initially introduced into spermatogonia. Our results strongly suggest that removal of B[a]PDE-N^2^-dG adduct is poor in the meiotic- and post-meiotic phases including spermiogenesis. This is also consistent with the dominant lethality observed in mice following B[a]P-exposure, which is most pronounced in the later stages of spermiogenesis but is also found in pre-leptotene spermatocytes [18]. B[a]PDE-N^2^-dG adducts are helix-distorting substrates for removal via NER. The results are hence in accordance with our previous findings, in rat male germ cells, of poor removal of other NER substrates such as UVC lesions and 2-acetylaminofluorene-adducts [16]; however, spermatogonia were not specifically studied. In mice, Xu and co-workers reported similar poor global genomic repair of cyclobutane pyrimidine dimers (CPDs) in male germ cells; however, repair of 6-4-photoproducts (6-4PP) was observed (using much higher UV doses than in our study [16]) although at markedly lower rates than in somatic cells [28]. Support for active NER in spermatogonia of mice is provided by the observation of moderate global genomic repair of 6-4PP and also strand-independent gene-specific repair of CPDs [28]. Taken together, NER in meiotic- and post-meiotic male germ cells appears to depend on a number of factors such as the species, the type of DNA adduct, the gene, and the exposure level.

The high level of B[a]PDE-N^2^-dG adducts in caput spermatozoa (76% of that observed in liver DNA; Figure 2) originating from epididymal spermatozoa collected at day 4 (Figure 1, Supplementary Table S1), strongly suggests that even densely packed spermatozoa are susceptible to induction of DNA adducts. Recently Verhofstad et al. [27] and Sipinen et al. [29] reported induction of significant levels of B[a]P-adducts in spermatozoa, using ^32^P-postlabeling and immunological techniques, in complete agreement with our present data in which we now demonstrate the chemical identity of the adducts (Figure 1, Supplementary Table S1). These data correspond to parallel findings in spermatozoa of human smokers showing increased adduct levels detected with immunological methods [30].

In conclusion, B[a]PDE-N^2^-dG adducts initially induced in spermatogonia do not persist to the stage of caput spermatozoa; they are likely to be removed by one or several mechanisms, namely DNA repair, cell death, or dilution by replication and cell division. Adducts formed in meiotic, post-meiotic and spermiogenic stages, on the other hand, persist in spermatozoa. The genome of the latter stages is therefore susceptible to at least some types of genotoxic insult, despite protective factors such as the blood-testis barrier and compaction of chromatin.

### *De novo* mutations

Mutation analysis using λ phage-based *cII* and *lacI/lacZ*-assays is limited to non-transcribed sequences and detection of base mutations and small insertions/deletions. When studying paternal mutations, *cII* and *lacI/lacZ* are suitable targets since paternal mutations most often are base mutations and small insertions/deletions. Furthermore these genes are non-transcribed and during spermatogenesis transcription ceases for a number of genes. B[a]P-exposure of stem cell spermatogonia gave rise to two-fold increased levels of *de novo cII*-mutations, present in cauda spermatozoa (Figure 3, Table 1). There seemed to be increased levels also following exposure of the other stages of spermatogenesis tested. The increased Mf in cauda spermatozoa originating from exposed elongating spermatids is highly unlikely to represent *de novo* germ line mutations since these cells do not replicate or repair their DNA. They are more likely to be derived from a low percentage of somatic cells present in the cauda spermatozoa preparations, or by post-packaging mutation generation in *E. coli* in the *cII*-selection system due to the presence of DNA adducts. The higher and more variable mean Mf for untreated mice, compared to the 119 days corn oil-mice, may be caused by the fact that the untreated mice are not littermates. The corn oil-treated and the B[a]P-exposed mice, on the other hand, were mostly littermates and were also age-matched. Lower variations within the group are expected among littermates, leading to more evident treatment-related differences. The statistically significant induction of *de novo* mutations in stem cell spermatogonia is in agreement with their active proliferation, which is important for mutation fixation but which is discontinued in spermatocytes. In the meiotic and post-meiotic cells, mutation fixation is limited to DNA synthesis associated with DNA recombination or repair. In comparison, both the spontaneous and the B[a]P-induced Mf were approximately two-fold higher in the liver (Figure 4, Table 2) than in spermatozoa (Figure 3, Table 1), as expected [31].

Other types of mutations, such as hypervariable expanded simple tandem repeat (ESTR), have been used to reveal male germ line *de novo* mutations. ESTR mutations are elevated in the germ line of untreated offspring from male mice exposed to ionizing radiation, at comparable to directly exposed males [32], and also in the second generation of male offspring [33]. Similarly, a first generation transmittance of increased ESTR mutation levels was observed in mice exposed to cigarette smoke [34] and particulate air pollution [35]. These increases all originate from and are restricted to exposed spermatogonia, in agreement with our present findings. Our study now demonstrates - in a non-transcribed gene-that B[a]P-exposure gives rise to a similar stem cell spermatogonia derived increase in *de novo* mutations which is measurable in the resulting spermatozoa. For spermatozoa containing DNA adducts, several mechanisms exist to prevent transmission of their genome to the offspring; such mechanisms are not known to exist for mutations in spermatozoa.

We report for the first time DNA sequence data in spermatozoa following *in vivo* exposure to B[a]P. Characterisation of the mutations from exposed spermatogonia showed increased levels of GC-TA transversions (1.5-1.9-fold; Table 2) being the signature mutation following B[a]P-exposure [36], [37]. Similarly, the induction of GC-TA was higher (3.3-fold) in the liver, as also reported by others [38].

Differences between the organs with respect to the spontaneous mutation patterns were observed. Spermatozoa exhibited a unique pattern of mutations compared to the liver. A markedly higher frequency of InsGs and also a higher frequency of GC-TA transversions along with a concomitant lower frequency of CG-TA transitions were found in cauda spermatozoa compared to the liver, suggesting that the spontaneous mutagenic process in the male germ line may be different from that of somatic cells. The difference was most evident prior to exclusion of potentially clonal mutants with spermatozoa exhibiting markedly higher numbers of InsG than the liver. The differences in InsG frequencies are not apparent after clonal exclusion since the frequency is greatly influenced by the numbers of animals analysed rather than the frequency of the mutations; most mice had InsG in the spermatozoa and only a few had InsG in their livers. The difference in InsG frequency may be even more pronounced since the exclusion of InsG at identical positions in the *cII*-gene as well as in the G-run probably leads to an underestimation of the actual frequency of InsG in spermatozoa.

As in most tissues, CG-TA transitions predominate also in spermatozoa; these are believed to originate from deamination of C or methylated C, of which a majority occurs in CpG dinucleotides. Using *lacZ* as a target gene, Douglas and co-workers similarly reported a high frequency of GC-TA transversions and low frequency of CG-TA transitions in male germ cells, compared to somatic tissues [39], although clonal mutations could not be excluded. However, *lacZ* does not contain a similar run of six guanines and increased frequencies of InsG are thus unlikely. The high frequency of InsG in longer runs of guanines observed in our study suggests that such sequence domains may be particularly sensitive to insertions in the mouse male germ line. The very low level of InsGs (Table 4) and clonally excluded InsGs (Table 3) among the liver *cII*-mutants in the same individuals substantiate the notion that the mutagenic process may be unique to the male germ line. Walter and co-workers observed a similar high frequency of InsGs among the spontaneous mutations of isolated mouse spermatogonia in the *lacI* gene [40]. Previous characterisations of spontaneous mutation patterns in *cII* in different somatic tissues have given variable results with respect to the G-run [41]–[44].

### Concluding remarks

Our results correspond with several observations in humans. Spermatozoa from smokers contain higher levels of DNA damage compared to non-smokers [3], [4], [45]. B[a]PDE derived adducts are present in spermatozoa of smoking men [30]. The DNA adducts are transmitted into the zygote via the male genome and are present in early human embryos [46]. Moreover, smoking reduces both the production of sperm, sperm quality and fecundity [47]–[49]. An association of sperm DNA damage and pregnancy loss following *in vitro* fertilization has been observed by several authors [50]–[55]. Furthermore, paternal smoking and exposure to polycyclic aromatic hydrocarbons significantly increase the risk of childhood cancer in offspring [56]–[61]. DNA adducts in spermatozoa therefore have several important implications.

This study combines analyses of stage-specific male germ-line *de novo* mutations, as well as a quantification and characterization of the pre-mutagenic lesions involved. We provide evidence that DNA adducts induced in male germ cells persist in spermatozoa and that they give rise to *de novo* mutations, also demonstrating B[a]P as a mouse germ cell mutagen. The mutation pattern of the spermatozoa suggests a unique mutagenic process in the male germ line both spontaneously and following B[a]P-exposure. Exposure to environmental, lifestyle and dietary derived agents during everyday life may thus have implications for the ability of men to conceive and potentially also for the health of their offspring.

## Supporting Information

Figure S1 Relative bodyweights and relative weights of reproductive organs of mice exposed to B[a]P. The values are given as means±SE percent of (A) the relative bodyweights (g/g), (B) relative testes weights (mg/g), and (C) relative epididymal weights (mg/g) of B[a]P- and corn oil-exposed mice, relative to untreated mice. The x-axes represent the day of sacrifice (A, untreated mice being sacrificed at day 0), and exposed germ cell stage (day of sacrifice after exposure) (B and C, cauda spermatozoa = CASZ representing untreated mice). The male germ cell stages are indicated with cauda spermatozoa = CASZ, epididymal spermatozoa = ESZ, spermatids = ST, spermatocytes = SC, differentiating spermatogonia = Diff. SG and stem cell spermatogonia = Stem cell SG. Statistical differences between B[a]P- and untreated mice (A and B) and between B[a]P- and corn oil-exposed mice (B and C) are indicated when p<0.05 (A: Mann-Whitney; B: ANOVA and post hoc test; C: Mann-Whitney).
(1.38 MB TIF)

Table S1 B[a]PDE-N^2^-dG adducts in caput spermatozoa.
(0.09 MB DOC)

Table S2 B[a]PDE-N^2^-dG adducts in liver.
(0.09 MB DOC)

## References

[1] Hull MG, Glazener CM, Kelly NJ, Conway DI, Foster PA (1985). Population study of causes, treatment, and outcome of infertility.. Br Med J (Clin Res Ed).

[2] Andersson AM, Jorgensen N, Main KM, Toppari J, Rajpert-De ME (2008). Adverse trends in male reproductive health: we may have reached a crucial ‘tipping point’.. Int J Androl.

[3] Fraga CG, Motchnik PA, Shigenaga MK, Helbock HJ, Jacob RA (1991). Ascorbic acid protects against endogenous oxidative DNA damage in human sperm.. Proc Natl Acad Sci U S A.

[4] Fraga CG, Motchnik PA, Wyrobek AJ, Rempel DM, Ames BN (1996). Smoking and low antioxidant levels increase oxidative damage to sperm DNA.. Mutat Res.

[5] Ni ZY, Liu YQ, Shen HM, Chia SE, Ong CN (1997). Does the increase of 8-hydroxydeoxyguanosine lead to poor sperm quality?. Mutat Res.

[6] Shen H, Ong C (2000). Detection of oxidative DNA damage in human sperm and its association with sperm function and male infertility.. Free Radic Biol Med.

[7] Irvine DS, Twigg JP, Gordon EL, Fulton N, Milne PA (2000). DNA integrity in human spermatozoa: relationships with semen quality.. J Androl.

[8] Schmid TE, Kamischke A, Bollwein H, Nieschlag E, Brinkworth MH (2003). Genetic damage in oligozoospermic patients detected by fluorescence in-situ hybridization, inverse restriction site mutation assay, sperm chromatin structure assay and the Comet assay.. Hum Reprod.

[9] Loft S, Kold-Jensen T, Hjollund NH, Giwercman A, Gyllemborg J (2003). Oxidative DNA damage in human sperm influences time to pregnancy.. Hum Reprod.

[10] Ahmadi A, Ng SC (1999). Fertilizing ability of DNA-damaged spermatozoa.. J Exp Zool.

[11] Nelson K, Holmes LB (1989). Malformations due to presumed spontaneous mutations in newborn infants.. N Engl J Med.

[12] Crow JF (2000). The origins, patterns and implications of human spontaneous mutation.. Nat Rev Genet.

[13] Vogel F, Rathenberg R (1975). Spontaneous mutation in man.. Adv Hum Genet.

[14] Stenson PD, Ball EV, Mort M, Phillips AD, Shiel JA (2003). Human Gene Mutation Database (HGMD): 2003 update.. Hum Mutat.

[15] Makova KD, Yang S, Chiaromonte F (2004). Insertions and deletions are male biased too: a whole-genome analysis in rodents.. Genome Res.

[16] Jansen J, Olsen AK, Wiger R, Naegeli H, de BP (2001). Nucleotide excision repair in rat male germ cells: low level of repair in intact cells contrasts with high dual incision activity in vitro.. Nucleic Acids Res.

[17] Olsen AK, Lindeman B, Wiger R, Duale N, Brunborg G (2005). How do male germ cells handle DNA damage?. Toxicol Appl Pharmacol.

[18] Generoso WM, Cain KT, Hellwig CS, Cacheiro NL (1982). Lack of association between induction of dominant-lethal mutations and induction of heritable translocations with benzo[a]pyrene in postmeiotic germ cells of male mice.. Mutat Res.

[19] Evenson DP, Baer RK, Jost LK (1989). Long-term effects of triethylenemelamine exposure on mouse testis cells and sperm chromatin structure assayed by flow cytometry.. Environ Mol Mutagen.

[20] Oskam IC, Lyche JL, Krogenaes A, Thomassen R, Skaare JU (2005). Effects of long-term maternal exposure to low doses of PCB126 and PCB153 on the reproductive system and related hormones of young male goats.. Reproduction.

[21] Evenson DP, Jost LK, Baer RK (1993). Effects of methyl methanesulfonate on mouse sperm chromatin structure and testicular cell kinetics.. Environ Mol Mutagen.

[22] Suter L, Koch E, Bechter R, Bobadilla M (1997). Three-parameter flow cytometric analysis of rat spermatogenesis.. Cytometry.

[23] Singh R, Gaskell M, Le Pla RC, Kaur B, Azim-Araghi A (2006). Detection and quantitation of benzo[a]pyrene-derived DNA adducts in mouse liver by liquid chromatography-tandem mass spectrometry: comparison with 32P-postlabeling.. Chem Res Toxicol.

[24] Andreassen A, Vikse R, Mikalsen A, Adamovic T, Steffensen IL (2006). 2-Amino-1-methyl-6-phenylimidazo[4,5-b]pyridine (PhIP) induces genetic changes in murine intestinal tumours and cells with ApcMin mutation.. Mutat Res.

[25] Adams WT, Skopek TR (1987). Statistical test for the comparison of samples from mutational spectra.. J Mol Biol.

[26] Meistrich ML (1986). Critical components of testicular function and sensitivity to disruption.. Biol Reprod.

[27] Verhofstad N, van Oostrom CT, van BJ, Van Schooten FJ, van SH (2009). DNA adduct kinetics in reproductive tissues of DNA repair proficient and deficient male mice after oral exposure to benzo(a)pyrene.. Environ Mol Mutagen.

[28] Xu G, Spivak G, Mitchell DL, Mori T, McCarrey JR (2005). Nucleotide excision repair activity varies among murine spermatogenic cell types.. Biol Reprod.

[29] Sipinen V, Laubenthal J, Baumgartner A, Cemeli E, Linschooten JO (2010). In vitro evaluation of baseline and induced DNA damage in human sperm exposed to benzo[a]pyrene or its metabolite benzo[a]pyrene-7,8-diol-9,10-epoxide, using the comet assay.. Mutagenesis.

[30] Zenzes MT, Bielecki R, Reed TE (1999). Detection of benzo(a)pyrene diol epoxide-DNA adducts in sperm of men exposed to cigarette smoke.. Fertil Steril.

[31] Kohler SW, Provost GS, Fieck A, Kretz PL, Bullock WO (1991). Analysis of spontaneous and induced mutations in transgenic mice using a lambda ZAP/lacI shuttle vector.. Environ Mol Mutagen.

[32] Dubrova YE, Jeffreys AJ, Malashenko AM (1993). Mouse minisatellite mutations induced by ionizing radiation.. Nat Genet.

[33] Barber R, Plumb MA, Boulton E, Roux I, Dubrova YE (2002). Elevated mutation rates in the germ line of first- and second-generation offspring of irradiated male mice.. Proc Natl Acad Sci U S A.

[34] Yauk CL, Berndt ML, Williams A, Rowan-Carroll A, Douglas GR (2007). Mainstream tobacco smoke causes paternal germ-line DNA mutation.. Cancer Res.

[35] Yauk C, Polyzos A, Rowan-Carroll A, Somers CM, Godschalk RW (2008). Germ-line mutations, DNA damage, and global hypermethylation in mice exposed to particulate air pollution in an urban/industrial location.. Proc Natl Acad Sci U S A.

[36] Carothers AM, Grunberger D (1990). DNA base changes in benzo[a]pyrene diol epoxide-induced dihydrofolate reductase mutants of Chinese hamster ovary cells.. Carcinogenesis.

[37] Ruggeri B, DiRado M, Zhang SY, Bauer B, Goodrow T (1993). Benzo[a]pyrene-induced murine skin tumors exhibit frequent and characteristic G to T mutations in the p53 gene.. Proc Natl Acad Sci U S A.

[38] Shane BS, de BJ, Watson DE, Haseman JK, Glickman BW (2000). LacI mutation spectra following benzo[a]pyrene treatment of Big Blue mice.. Carcinogenesis.

[39] Douglas GR, Gingerich JD, Gossen JA, Bartlett SA (1994). Sequence spectra of spontaneous lacZ gene mutations in transgenic mouse somatic and germline tissues.. Mutagenesis.

[40] Walter CA, Intano GW, McMahan CA, Kelner K, McCarrey JR (2004). Mutation spectral changes in spermatogenic cells obtained from old mice.. DNA Repair (Amst).

[41] Harbach PR, Zimmer DM, Filipunas AL, Mattes WB, Aaron CS (1999). Spontaneous mutation spectrum at the lambda cII locus in liver, lung, and spleen tissue of Big Blue transgenic mice.. Environ Mol Mutagen.

[42] Andrew SE, Xu XS, Baross-Francis A, Narayanan L, Milhausen K (2000). Mutagenesis in.. Carcinogenesis.

[43] Yoon JH, Smith LE, Feng Z, Tang M, Lee CS (2001). Methylated CpG dinucleotides are the preferential targets for G-to-T transversion mutations induced by benzo[a]pyrene diol epoxide in mammalian cells: similarities with the p53 mutation spectrum in smoking-associated lung cancers.. Cancer Res.

[44] Yoon JH, Besaratinia A, Feng Z, Tang MS, Amin S (2004). DNA damage, repair, and mutation induction by (+)-Syn and (-)-anti-dibenzo[a,l]pyrene-11,12-diol-13,14-epoxides in mouse cells.. Cancer Res.

[45] Shen HM, Chia SE, Ni ZY, New AL, Lee BL (1997). Detection of oxidative DNA damage in human sperm and the association with cigarette smoking.. Reprod Toxicol.

[46] Zenzes MT, Puy LA, Bielecki R, Reed TE (1999). Detection of benzo[a]pyrene diol epoxide-DNA adducts in embryos from smoking couples: evidence for transmission by spermatozoa.. Mol Hum Reprod.

[47] Ramlau-Hansen CH, Thulstrup AM, Aggerholm AS, Jensen MS, Toft G (2007). Is smoking a risk factor for decreased semen quality? A cross-sectional analysis.. Hum Reprod.

[48] Hassan MA, Killick SR (2004). Negative lifestyle is associated with a significant reduction in fecundity.. Fertil Steril.

[49] Hull MG, North K, Taylor H, Farrow A, Ford WC (2000). Delayed conception and active and passive smoking. The Avon Longitudinal Study of Pregnancy and Childhood Study Team.. Fertil Steril.

[50] Evenson DP, Jost LK, Marshall D, Zinaman MJ, Clegg E (1999). Utility of the sperm chromatin structure assay as a diagnostic and prognostic tool in the human fertility clinic.. Hum Reprod.

[51] Borini A, Tarozzi N, Bizzaro D, Bonu MA, Fava L (2006). Sperm DNA fragmentation: paternal effect on early post-implantation embryo development in ART.. Hum Reprod.

[52] Lin MH, Kuo-Kuang LR, Li SH, Lu CH, Sun FJ (2008). Sperm chromatin structure assay parameters are not related to fertilization rates, embryo quality, and pregnancy rates in in vitro fertilization and intracytoplasmic sperm injection, but might be related to spontaneous abortion rates.. Fertil Steril.

[53] Zini A, Boman JM, Belzile E, Ciampi A (2008). Sperm DNA damage is associated with an increased risk of pregnancy loss after IVF and ICSI: systematic review and meta-analysis.. Hum Reprod.

[54] Zitzmann M, Rolf C, Nordhoff V, Schrader G, Rickert-Fohring M (2003). Male smokers have a decreased success rate for in vitro fertilization and intracytoplasmic sperm injection.. Fertil Steril.

[55] Frydman N, Prisant N, Hesters L, Frydman R, Tachdjian G (2008). Adequate ovarian follicular status does not prevent the decrease in pregnancy rates associated with high sperm DNA fragmentation.. Fertil Steril.

[56] Ji BT, Shu XO, Linet MS, Zheng W, Wacholder S (1997). Paternal cigarette smoking and the risk of childhood cancer among offspring of nonsmoking mothers.. J Natl Cancer Inst.

[57] Lee KM, Ward MH, Han S, Ahn HS, Kang HJ (2009). Paternal smoking, genetic polymorphisms in CYP1A1 and childhood leukemia risk.. Leuk Res.

[58] Sorahan T, Prior P, Lancashire RJ, Faux SP, Hulten MA (1997). Childhood cancer and parental use of tobacco: deaths from 1971 to 1976.. Br J Cancer.

[59] Cordier S, Lefeuvre B, Filippini G, Peris-Bonet R, Farinotti M (1997). Parental occupation, occupational exposure to solvents and polycyclic aromatic hydrocarbons and risk of childhood brain tumors (Italy, France, Spain).. Cancer Causes Control.

[60] Cordier S, Monfort C, Filippini G, Preston-Martin S, Lubin F (2004). Parental exposure to polycyclic aromatic hydrocarbons and the risk of childhood brain tumors: The SEARCH International Childhood Brain Tumor Study.. Am J Epidemiol.

[61] Plichart M, Menegaux F, Lacour B, Hartmann O, Frappaz D (2008). Parental smoking, maternal alcohol, coffee and tea consumption during pregnancy and childhood malignant central nervous system tumours: the ESCALE study (SFCE).. Eur J Cancer Prev.

