# Supplementary figures and images for: Environmental Exposure of the Mouse Germ Line: DNA Adducts in Spermatozoa and Formation of De Novo Mutations during Spermatogenesis
Source: PLoS One. 2010 Jun 28;5(6):e11349. doi: 10.1371/journal.pone.0011349 (PMC2893163; doi:10.1371/journal.pone.0011349)

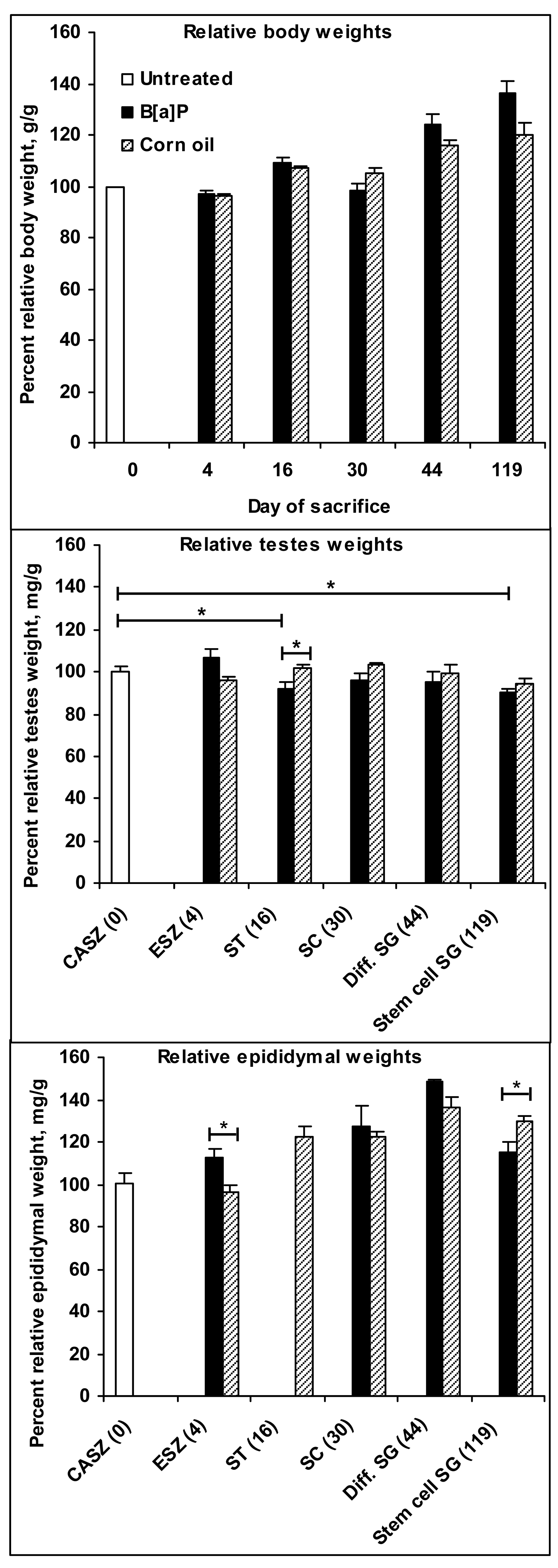

Supplement: Figure S1 — Relative bodyweights and relative weights of reproductive organs of mice exposed to B[a]P. The values are given as means±SE percent of (A) the relative bodyweights (g/g), (B) relative testes weights (mg/g), and (C) relative epididymal weights (mg/g) of B[a]P- and corn oil-exposed mice, relative to untreated mice. The x-axes represent the day of sacrifice (A, untreated mice being sacrificed at day 0), and exposed germ cell stage (day of sacrifice after exposure) (B and C, cauda spermatozoa = CASZ representing untreated mice). The male germ cell stages are indicated with cauda spermatozoa = CASZ, epididymal spermatozoa = ESZ, spermatids = ST, spermatocytes = SC, differentiating spermatogonia = Diff. SG and stem cell spermatogonia = Stem cell SG. Statistical differences between B[a]P- and untreated mice (A and B) and between B[a]P- and corn oil-exposed mice (B and C) are indicated when p<0.05 (A: Mann-Whitney; B: ANOVA and post hoc test; C: Mann-Whitney). (1.38 MB TIF) [file pone.0011349.s001.tif]
