# Supplementary material for: Environmental Exposure of the Mouse Germ Line: DNA Adducts in Spermatozoa and Formation of De Novo Mutations during Spermatogenesis
Source: PLoS One. 2010 Jun 28;5(6):e11349. doi: 10.1371/journal.pone.0011349 (PMC2893163; doi:10.1371/journal.pone.0011349)
Supplement: Table S1 — B[a]PDE-N2-dG adducts in caput spermatozoa. (0.09 MB DOC) [file pone.0011349.s002.doc]

**Table S2 B[a]PDE-N^2^-dG adducts in caput spermatozoa.**

| **Caput spermatozoa** | | | | | | | |
| --- | --- | --- | --- | --- | --- | --- | --- |
| **Day of sacrifice** | **Treatment** | **n** | **Amount of DNA analysed (µg)** | **B[a]PDEdG (fmol)** | **B[a]PDEdG adducts per 10^8^ deoxynucleotides** | **Mean** | **SE** |
| 0 | None | 4 | 45.0 | nd | nd | nd |  |
|  |  |  | 39.2 | nd | nd |  |  |
|  |  |  | 45.0 | nd | nd |  |  |
|  |  |  | 45.0 | nd | nd |  |  |
| 4 | B[a]P | 4 | 34.2 | 95.6 | 86.3 | 87.4 | 8.9 |
|  |  |  | 45.0 | 80.7 | 66.4 |  |  |
|  |  |  | 45.0 | 110.5 | 110.0 |  |  |
|  |  |  | 45.0 | 76.4 | 87.0 |  |  |
| 16 | B[a]P | 4 | 27.8 | 77.5 | 86.0 | 40.7* | 15.2 |
|  |  |  | 32.3 | 23.4 | 22.3 |  |  |
|  |  |  | 50.0 | 46.9 | 28.9 |  |  |
|  |  |  | 50.0 | 41.4 | 25.6 |  |  |
| 30 | B[a]P | 6 | 45.0 | 37.8 | 40.6 | 28.5** | 3.8 |
|  |  |  | 45.0 | 23.6 | 17.9 |  |  |
|  |  |  | 27.2 | 31.0 | 35.2 |  |  |
|  |  |  | 50.0 | 33.0 | 20.4 |  |  |
|  |  |  | 50.0 | 37.4 | 23.1 |  |  |
|  |  |  | 45.0 | 25.2 | 33.9 |  |  |
| 44 | B[a]P | 4 | 45.0 | nd | nd | nd |  |
|  |  |  | 45.0 | nd | nd |  |  |
|  |  |  | 45.0 | nd | nd |  |  |
|  |  |  | 22.1 | nd | nd |  |  |
| 119 | B[a]P | 5 | 45.0 | nd | nd | nd |  |
|  |  |  | 31.7 | nd | nd |  |  |
|  |  |  | 45.0 | nd | nd |  |  |
|  |  |  | 45.0 | nd | nd |  |  |
|  |  |  | 45.0 | nd | nd |  |  |
| 119 | Corn oil | 5 | 41.2 | nd | nd | nd |  |
|  |  |  | 45.0 | nd | nd |  |  |
|  |  |  | 45.0 | nd | nd |  |  |
|  |  |  | 45.0 | nd | nd |  |  |
|  |  |  | 45.0 | nd | nd |  |  |

nd = none detected (below detection level)

* = Significantly different (p=0.006, ANOVA and post hoc LSD) compared to the mean adduct level at 4 days after B[a]P-exposure

** = Significantly different (p=0.001, ANOVA and post hoc LSD) compared to the mean adduct level at 4 days after B[a]P-exposure
