# Supplementary material for: Environmental Exposure of the Mouse Germ Line: DNA Adducts in Spermatozoa and Formation of De Novo Mutations during Spermatogenesis
Source: PLoS One. 2010 Jun 28;5(6):e11349. doi: 10.1371/journal.pone.0011349 (PMC2893163; doi:10.1371/journal.pone.0011349)
Supplement: Table S2 — B[a]PDE-N2-dG adducts in liver. (0.09 MB DOC) [file pone.0011349.s003.doc]

**Table S3. B[a]PDE-N^2^-dG adducts in liver.**

| **Liver** | | | | | | | |
| --- | --- | --- | --- | --- | --- | --- | --- |
| **Day of sacrifice** | **Treatment** | **n** | **Amount of DNA analysed (µg)** | **B[a]PDEdG**  **(fmol)** | **B[a]PDEdG adducts per 10^8^ deoxynucleotides** | **Mean** | **SE** |
| 0 | None | 4 | 45.0 | nd | nd | nd |  |
|  |  |  | 45.0 | nd | nd |  |  |
|  |  |  | 45.0 | nd | nd |  |  |
|  |  |  | 45.0 | nd | nd |  |  |
| 4 | B[a]P | 4 | 45.0 | 211.4 | 145.0 | 115.5 | 11.2 |
|  |  |  | 45.0 | 141.2 | 96.8 |  |  |
|  |  |  | 45.0 | 175.5 | 120.4 |  |  |
|  |  |  | 45.0 | 145.3 | 99.7 |  |  |
| 16 | B[a]P | 4 | 50.0 | 47.9 | 29.5 | 26.0* | 2.5 |
|  |  |  | 50.0 | 32.8 | 20.3 |  |  |
|  |  |  | 27.1 | 27.0 | 30.8 |  |  |
|  |  |  | 28.0 | 21.6 | 23.2 |  |  |
| 30 | B[a]P | 8 | 45.0 | 37.4 | 25.6 | 15.4** | 4.1 |
|  |  |  | 45.0 | nd | nd |  |  |
|  |  |  | 45.0 | 37.6 | 25.7 |  |  |
|  |  |  | 45.0 | nd | nd |  |  |
|  |  |  | 50.0 | 32.6 | 20.1 |  |  |
|  |  |  | 50.0 | 22.1 | 13.7 |  |  |
|  |  |  | 38.0 | 11.1 | 9.0 |  |  |
|  |  |  | 32.7 | 30.9 | 29.2 |  |  |
| 44 | B[a]P | 4 | 45.0 | nd | nd | 3.7***,† | 3.7 |
|  |  |  | 45.0 | nd | nd |  |  |
|  |  |  | 45.0 | nd | nd |  |  |
|  |  |  | 45.0 | 20.6 | 14.6 |  |  |
| 119 | B[a]P | 5 | 45.0 | nd | nd | nd |  |
|  |  |  | 45.0 | nd | nd |  |  |
|  |  |  | 45.0 | nd | nd |  |  |
|  |  |  | 45.0 | nd | nd |  |  |
|  |  |  | 45.0 | nd | nd |  |  |
| 119 | Corn oil | 5 | 45.0 | nd | nd | nd |  |
|  |  |  | 45.0 | nd | nd |  |  |
|  |  |  | 45.0 | nd | nd |  |  |
|  |  |  | 45.0 | nd | nd |  |  |
|  |  |  | 45.0 | nd | nd |  |  |

nd = none detected (below detection level)

* = Significantly different (p=0.021, Mann-Whitney) compared to the mean adduct level at 4 days after B[a]P-exposure

** = Significantly different (p=0.006, Mann-Whitney) compared to the mean adduct level at 4 days after B[a]P-exposure

*** = Significantly different (p=0.018, Mann-Whitney) compared to the mean adduct level at 4 days after B[a]P-exposure

† = Significantly different (p=0.018, Mann-Whitney) compared to the mean adduct level at 16 days after B[a]P-exposure
